# Optimization of Sputtering Process for Medium Entropy Alloy Nanotwinned CoCrFeNi Thin Films by Taguchi Method

**DOI:** 10.3390/ma15228238

**Published:** 2022-11-20

**Authors:** Jing-Yi Zhong, Jian-Jie Wang, Fan-Yi Ouyang

**Affiliations:** 1 Department of Engineering and System Science, National Tsing Hua University, Hsinchu 300044, Taiwan; 2 High Entropy Materials Center, National Tsing Hua University, Hsinchu 300044, Taiwan

**Keywords:** medium entropy alloys, nanotwinned structure, magnetron sputtering, Taguchi method, CoCrFeNi thin films

## Abstract

We demonstrate a systematic study optimizing the properties of CoCrFeNi medium entropy alloy (MEA) thin films by tuning the deposition parameters of the pulsed direct current (DC) magnetron sputtering process. The chemical composition and microstructure of thin films were studied with energy dispersive X-ray spectroscopy (EDS), an X-ray diffractometer (XRD) and a transmission electron microscope (TEM). Abundant nanotwins and the dual face-centered cubic−hexagonal close-packed (FCC-HCP) phases were formed in some specimens. The Taguchi experimental method and analysis of variance (ANOVA) were applied to find the optimized parameters. The control factors are five deposition parameters: substrate bias, substrate temperature, working pressure, rotation speed and pulsed frequency. According to the signal-to-noise ratio results, the optimized parameters for low electrical resistivity (98.2 ± 0.8 μΩ·cm), low surface roughness (0.5 ± 0.1 nm) and high hardness (9.3 ± 0.2 GPa) were achieved and verified with confirmed experiments.

## 1. Introduction

Different from conventional alloy designs, medium entropy alloys (MEAs) and high entropy alloys (HEAs) lead alloy design to a new stage [1]. Bulk MEAs and HEAs have attracted much interest and been widely investigated due to their superior properties, such as high hardness, superior corrosion resistance and thermal stability at elevated temperature [2,3,4,5,6,7,8,9,10,11]. Recently, MEA and HEA thin films have attracted a lot of attention due to their unique properties and wide application, such as in diffusion barriers and hard coatings [12,13,14,15,16,17].

Among plenty of MEAs and HEAs, CoCrFeNi-based alloys have attracted a lot of attention due to their interesting deformation behavior at high strain [18] and high toughness over a wide temperature range [5]. Within the constituent elements, Co is hard and resistant to high temperatures, but it is expensive. Although Cr is extremely useful for corrosion resistance, it can cause embrittlement when it forms a second phase [19]. Ni is thermally stable in a wide range of temperatures, while Fe has low-cost advantages. The mechanical properties and thermal stability of bulk CoCrFeNi have been explored by numerous researchers using various fabrication processes, such as cold drawing [20], arc melting [21], and high-pressure torsion [22]. However, studies have rarely been conducted on CoCrFeNi thin films. Furthermore, the hardness of the sputtered CoCrFeNi film (9.8 ± 0.3 GPa) was found to be higher than that of bulk CoCrFeNi prepared by high-pressure torsion (7.3 ± 0.3 GPa) [23]. Therefore, optimizing the property of CoCrFeNi thin films by tuning various sputtering parameters is important.

Deposition parameters have significant effects on the microstructure and properties of thin films. Khan et al. fabricated AlCoCrCu_0.5_FeNi by radio frequency (RF) magnetron sputtering with three different working pressures (5, 10 and 15 mTorr); they found that films deposited at 10 mTorr possessed the largest grain size and highest surface roughness [24]. Sha et al. deposited CoCrFeMnNi HEA coatings on M2 steel by direct current (DC) magnetron sputtering under different substrate bias voltages (−20, −60 and −120 V) [25]; a high hardness of ~9.1 GPa was achieved in the coatings deposited at −120 V. Lin et al. fabricated (Cr_0.35_Al_0.25_Nb_0.12_Si_0.08_V_0.20_) N HEA nitride films by RF magnetron sputtering, and the hardness of films can be improved from 28 GPa to 35 GPa by tuning substrate temperature and substrate bias [26]. Köçkar and Şentürk concluded that the rotation speed of the substrate plays a considerable role in the structural and magnetic properties of FeNiCrCd thin films [27]. Hsiao et al. found that the duty cycle of high-power impulse magnetron sputtering (HIPIMS) showed significant influence on the microstructure of AlCrN coatings [28]. In this study, the medium entropy alloy CoCrFeNi films were deposited by pulsed DC magnetron sputtering. The properties of CoCrFeNi films were optimized by adjusting five major deposition parameters: substrate bias, substrate temperature, working pressure, rotation speed of substrate and pulsed frequency. The Taguchi method and analysis of variance (ANOVA) were introduced to investigate the optimized parameter for high hardness, low surface roughness and low electrical resistivity of the films.

## 2. Experimental Methods

### 2.1. Sample Preparation

To fabricate the 3-inch quaternary CoCrFeNi targets, 99.99 wt.% purity Co, Cr, Fe, and Ni pellets were first melted by an arc under high-purity argon gas on a water-cooled Cu mold five times to achieve homogeneous distribution of the elements in the alloy. Then, a target with a diameter of 76.2 mm and a thickness of 6 mm was formed from the ingot. The composition of the alloy target was analyzed using an inductively coupled plasma-optical emission spectrometer (ICP-MS, Agilent 7500ce). Second, the films were deposited on Si by a pulsed direct current (DC) magnetron sputtering system. Before deposition, the P-type Si (100) substrate was ultrasonically cleaned by acetone and methanol for 5 min each. The samples were loaded into the chamber and the target was struck by ion bombardment under a voltage of −1000 V for 7 min to remove the surface oxide when the base pressure was lower than 4.0 × 10^−6^ torr. All the samples were deposited at a gun power of 150 W and a reverse time of 1.6 µs for 80 min. External cooling or heating was not used during deposition. Five deposition parameters, including (A) substrate bias (0, −50, −100 and −150 V), (B) substrate temperature (room temperature (RT), 150, 250 and 350 °C), (C) working pressure (1.6, 1.4, 1.1 and 0.9 mTorr), (D) rotation speed of substrate (11, 24, 34 and 43 rpm) and (E) pulsed frequency (20, 60, 120 and 250 kHz), were set as control factors. The levels of the control factors and the deposition parameters for each sample are listed in Table 1 and Table 2, respectively.

### 2.2. S/N Ratio Analysis and Analysis of Variance (ANOVA)

The Taguchi method uses signal-to-noise ratio (S/N ratio) (unit: dB) to define the product quality.

(1) $$\;SN=-10log\left(\frac{1}{n}\sum_{i=1}^{n}\frac{1}{y_{i}{}^{2}}\right)\;\mathrm{Larger}\;\mathrm{the}\;\mathrm{better}$$

(2) $$SN=-10log\left(\frac{1}{n}\sum_{i=1}^{n}y_{i}{}^{2}\right)\;\mathrm{Smaller}\;\mathrm{the}\;\mathrm{better}$$

where $y_{i}$ is the value of the ith experiment and *n* is the total number of experiments. For film hardness, the S/N ratio was calculated using Equation (1), while for surface roughness and resistivity, the S/N ratio was calculated using Equation (2). Analysis of variance (ANOVA) was applied to the S/N ratio to quantify the influence of different sputter conditions on film properties. In the analysis, several terms are introduced, including degree of freedom (DOF), sum of squares (SS), contribution, mean square deviation (MS) and F [29].

Take an L_16_ orthogonal (Table 2) for example; the effect of parameter A at level 1 ($SN_{A1}$) can be estimated by

(3) $$SN_{A1}=\frac{1}{4}\left(SN_{1}+SN_{2}+SN_{3}+SN_{4}\right)$$

where $SN_{i}$ represents the S/N ratio of the ith row. The overall mean of the S/N ratio ($\underline{SN}$) can be calculated by

(4) $$\underline{SN}=\frac{1}{16}\;\sum_{1}^{16}SN_{i}$$

Then, the prediction value ($SN_{opt}$) can be calculated by

(5) $$SN_{opt}=\underline{SN}+\left(SN_{x}-\underline{SN}\right)+(SN_{y}-\underline{SN})$$

where $\underline{SN}$ is the mean value of S/N ratio, and $SN_{x}$ and $SN_{y}$ are the mean effects of sensitive parameters at the optimal level. The confidence interval of the prediction value can be calculated by

(6) $$CL_{opt}=SN_{opt}\pm \sqrt{F\left(1,n_{2}\right)\times MS_{e}/N_{e}}$$

where $F\left(1,n_{2}\right)$ is the value from the F-table at a required confidence level and at $DOF_{1}=1$ and $DOF_{2}=degree\;of\;freedoms\;of\;error$, $MS_{e}$ is the mean square deviation of error derived from ANOVA, and the equivalent sample size ($N_{e})$ can be calculated using the following equation:

(7) $$N_{e}=\frac{n}{DOF\;of\;mean\;\left(=1\right)+DOF\;of\;all\;factors\;included\;in\;the\;estimate\;of\;the\;mean}$$

where n is the total trials. From the process above, the prediction values at optimized parameters and its confidence interval can be derived. Confirmation tests were conducted, and the experiment value was compared with the prediction value to verify the Taguchi experiment.

### 2.3. Characterization Methods

After deposition, the elemental composition of the films was obtained by scanning electron microscopy-energy dispersive X-ray spectroscopy (SEM-EDS, JSM-7610F, JEOL, Tokyo, Japan), averaged from 5 regions of the film. The X-ray diffractometer (XRD, Bruker D2 PHASER for the θ/2θ scan and Bruker D8 DISCOVER for the grazing incidence scan, Bruker, Billerica, USA) was used to identify the crystal structure and preferred orientation. Grain size can be calculated using the Scherrer equation:

(8) $$\beta_{sample}=\frac{K\lambda }{dcos\theta }$$

where *β* is the full width at half maximum (FWHM) of the diffraction peak, K is the constant (shape factor) of 0.94 for a cubic crystal, d is the grain size, λ is the wavelength of Cu Kα radiation (0.154 nm) and *θ* is the peak position of (hkl) plane. The focused ion beam microscope (FIB, Helios NanoLab 600i, FEI, Hillsboro, USA) was used for TEM sample preparation and film thickness measurement. The average thickness of film was calculated based on FIB images from five different locations. A transmission electron microscope (TEM, JEM-F200, JEOL, Tokyo, Japan) was used for phase identification and observation of the cross-sectional microstructure. The residual stress was measured by a laser curvature system. Each sample was measured in three different directions to obtain an average residual stress. The residual stress was calculated by Stoney’s equation as follows [30]:

(9) $$\sigma_{f}=\frac{E_{s}t_{s}^{2}}{\left(1-\nu_{s}\right)6t_{f}}\left(\frac{1}{R}-\frac{1}{R_{0}}\right)$$

where $\sigma_{f}$ is the residual stress in the film; $t_{s}$ and $t_{f}$ are the thicknesses of the substrate and film, respectively; $E_{s}$ and $\nu_{s}$ are the Young’s modulus and Poisson’s ratio of the substrate, respectively; $R_{0}$ is the curvature of the substrate; and R is the curvature of the thin film. Here, $\frac{E_{s}}{1-\nu_{s}}$ equals 180 GPa for the Si substrate [31], and $t_{s}$ is 370 µm.

The electrical resistivity was measured by a four-point probe and can be calculated by

(10) $$\rho =\frac{V}{I}\times F\times t$$

where ρ is the sheet resistance, V is the voltage, I is the current, *F* is the correction factor related to aspect ratio and size of the sample, and t is the thickness of the film. The size of the sample is $25.4\times 25.4{\;\mathrm{mm}}^{2}$, so the correction factor is 4.31. A scanning probe microscope (SPM) with a tapping mode was used to measure the surface roughness. The film hardness was measured by nanoindentation (Hysitron Triboscop, Bruker, Billerica, USA) at room temperature. During the nanoindentation test, a Berkvovich diamond indenter with a nominal radius of 25 μm was used and a constant load of 2250 µN was applied, leading to a depth of displacement less than one-tenth of the film to avoid the substrate effect. Based on the measured loading/unloading curves of the nanoindentation test, the hardness was calculated using the analysis method proposed by Oliver and Pharr [32].

## 3. Results and Discussion

The 16 trials of experiments are denoted from S1 to S16, and the confirmed test for optimized electrical resistivity, surface roughness and hardness are denoted as SE, SR and SH, respectively.

### 3.1. Characteristics of CoCrFeNi Thin Films

The chemical compositions of Co, Cr, Fe and Ni in the medium entropy alloy target measured by ICP-MS are 25.4, 25.2, 24.6 and 24.8 at.%, respectively. The results demonstrate that the composition of the elements in the target is close to 25 at.% before melting, implying that the elements are well mixed in the target during the arc melting process.

**Figure 1:**
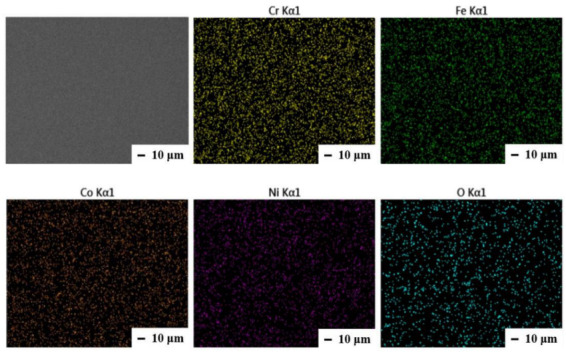
SEM image and EDS mapping results of an as-deposited film (S9) on Si substrate.

Table 3 shows the chemical composition of each film measured by EDS. As shown in Figure 1, no obvious segregation on the surface of films is observed, and uniform distribution of each element is achieved. The average concentration of oxygen in thin films is 2.3 ± 0.3%, which may be due to the residual gas in the chamber during the arc-melting process. The concentration of Cr was close to the designed value of the target, but the amount of Co in the films is slightly higher than that in the target. The amount of Fe and Ni slightly decreases when compared to target concentration.

Table 2 shows the thickness and deposition rate of each sample. The deposition rate was calculated based on thickness divided by the deposition time (80 min). The thickness of 16 samples ranges from 913.3 ± 8.9 nm to 1174.4 ± 13.0 nm. The average thickness is 1036.7 ± 16.6 nm, and the average deposition rate is 13.0 ± 0.2 nm/min. In general, the deposition rate decreases with higher negative bias due to the densification and re-sputtering effect.

**Figure 2:**
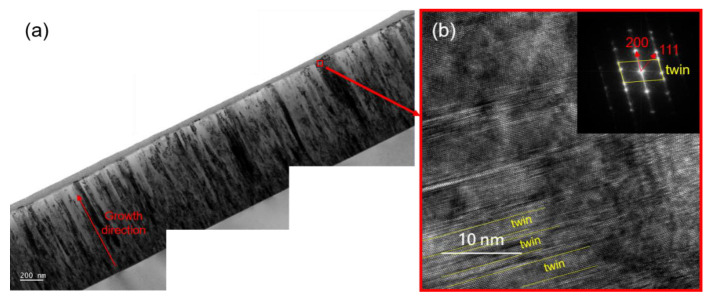
(**a**) The Cross-sectional TEM images of SH, and (**b**) HRTEM image of nanotwin in SH and the corresponding FFT pattern.

**Figure 3:**
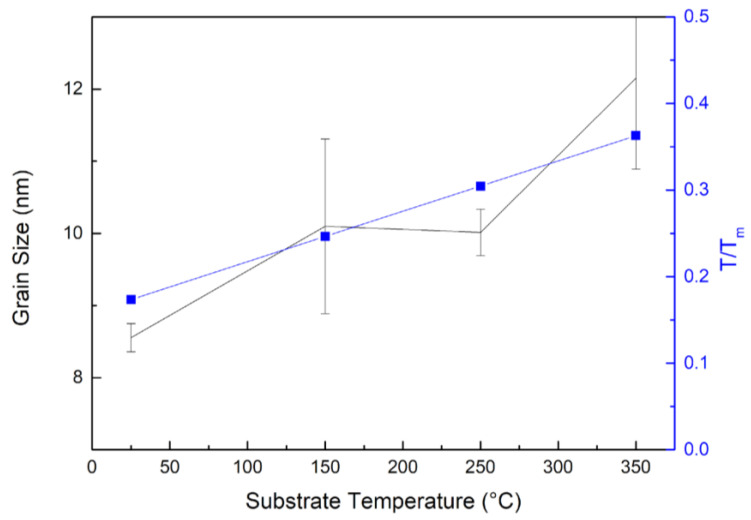
The relationship between grain size and substrate temperature (black line). The blue line indicates T/T_m_ in terms of substrate temperature, where T is substrate temperature and T_m_ is melting temperature of MEA.

The cross-sectional microstructure of CoCrFeNi thin film generally shows columnar grains with plenty planar defects, as shown in Figure 2a. According to the enlarged HRTEM image and the corresponding FFT pattern in Figure 2b, we can see the presence of a nanotwin in the SH sample. The dashed lines mark the location of the twins, and the average twin spacing is ~2 nm. In the inserted FFT image, the twin spots can also be observed. Table 2 summarizes the results calculated from the XRD analysis, including grain size, FWHM and lattice parameters. The average grain size of the 16 samples is 10.2 ± 2.3 nm, with a maximum of 15.6 nm (S12) and a minimum of 6.7 nm (S2). The relationship between the grain size and the temperature is displayed in Figure 3. The grain size of sample was observed to increase from 10.01 nm to 12.15 nm as temperature rises from 250 °C to 350 °C possibly due to the enhanced diffusivity at higher temperatures. These results are also consistent with the structural zone model (SZM) given that T/T_m_ = 0.30 at T = 250 °C and T/T_m_ = 0.36 at T = 350 °C.

**Figure 4:**
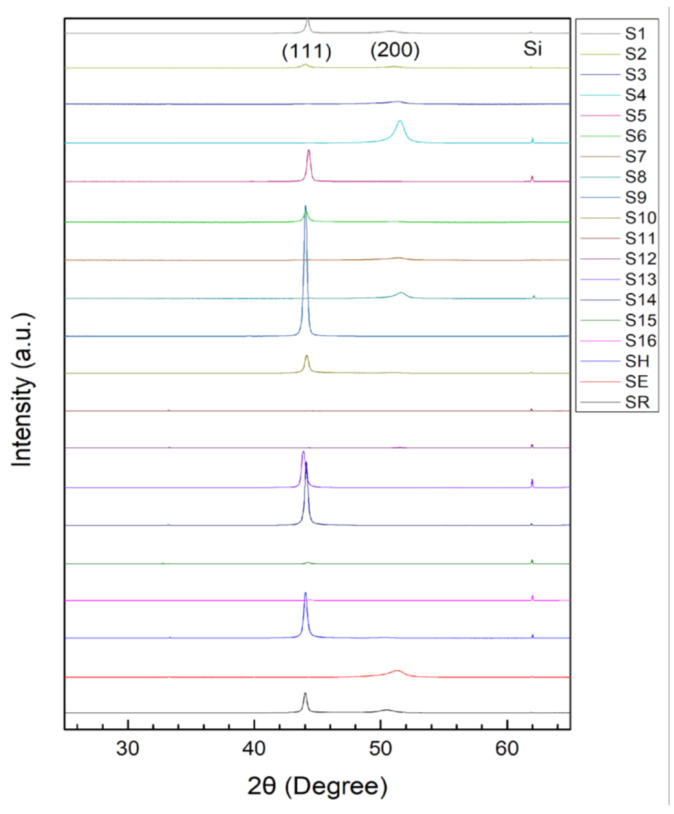
θ/2θ XRD patterns of each film.

**Figure 5:**
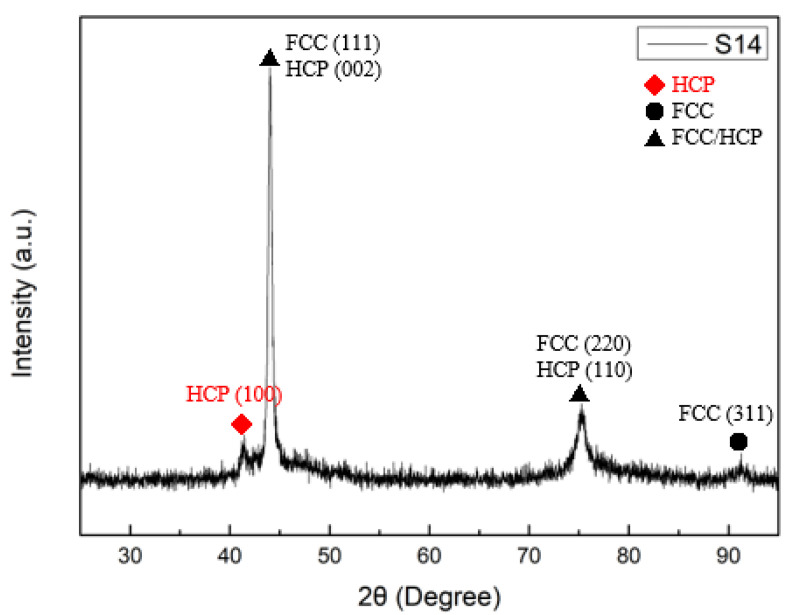
GIXRD diffraction pattern of S14.

**Figure 6:**
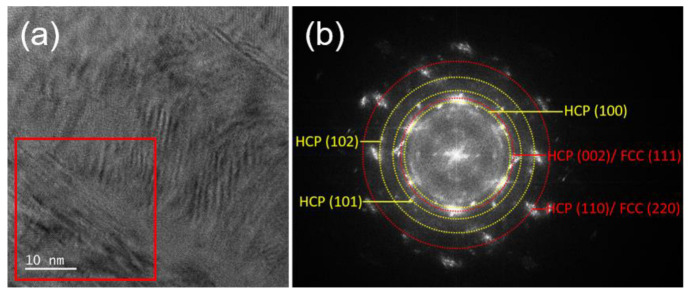
(**a**) HRTEM of S14; (**b**) the corresponding pattern of the red-square region on the left side.

The θ/2θ XRD patterns of the films are displayed in Figure 4; the XRD results show that CoCrFeNi has a major FCC phase. Because the valence electron concentration (VEC) of CoCrFeNi is 8.25, an FCC phase will form when the valence electron concentration (VEC) is ≥ 8 [33]. We also found that some samples show a dual-phase structure composed of a major FCC phase and a minor HCP phase. For example, HCP peaks occurred in S14, as shown in Figure 5. Since the peaks of FCC (111) and HCP (002) as well as the peaks of FCC (220) and HCP (110) are overlapped, TEM was introduced to analyze S14 to verify the existence of an HCP phase. Figure 6a is a high-resolution transmission electron microscopy (HRTEM) image of S14, and the fast Fourier transform (FFT) pattern of the red-square region is presented in Figure 6b. The yellow dash rings indicate HCP planes, confirming the existence of an HCP phase.

The lattice parameters of the samples estimated by the diffraction peaks in the GIXRD patterns are tabulated in Table 2. The average lattice parameter of the 16 samples was calculated to be a_FCC_ = 3.57 Å. In addition, for samples having an HCP phase, i.e., S14, the lattice parameters of the HCP phase were calculated as a_HCP_ = 2.53 Å and c_HCP_ = 4.10 Å, which are in agreement with those of the JCPD database.

Many studies have been theoretically investigated on the stacking fault energies (SFEs) of the HCP structure compared with that of the FCC structure in CoCrFeNi-group alloys. Zhang et al. found that the SFE of CoCrNi and CoCrFeNi could be significantly affected by local atomic distribution, temperature and severe lattice distortion. They also found that the HCP phase is thermodynamically more stable than the FCC phase at cryogenic temperatures [34]. Liu et al. performed first-principles calculations of CoCrFeNi with different configurations [35] and proved that the HCP phase has low energy. Nevertheless, most experimental results presented that CoCrFeNi contained a single phase of FCC because the FCC-to-HCP transformation requires the driving force to overcome the kinetic barrier [23,36,37]. The reason why the HCP phase was formed in this study may be due to the following reasons: First, the magnetron sputtering system provided sufficient energy for the films to overcome the barrier of FCC-to-HCP phase transformation. Second, additional bias applied on the substrate may attract more Ar^+^ ions for bombardment and it further provides energy to overcome the transformation barrier. These results were in accordance with the previous research conducted by Lin et al. [25]; the presence of partial FCC-HCP transformation was also found in a CoCrFeMnNi sputtered thin film with a higher negative substrate bias. The reasons above explain why the HCP phase was locally found in the samples in this study, especially those deposited under higher negative bias.

**Figure 7:**
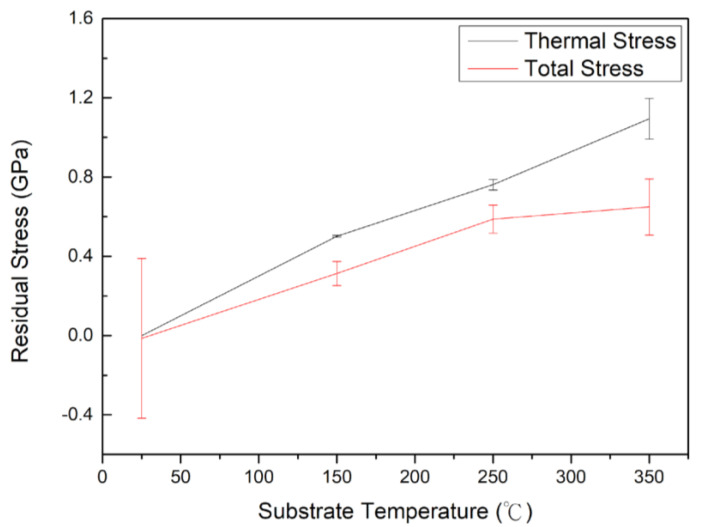
The residual stress of CoCrFeNi film as a function of substrate temperature.

Table 2 displays the residual stress of each film measured by laser curvature equipment. The residual stress of the 16 samples ranges from −1.22 ± 0.04 Pa to 0.98 ± 0.07 GPa. Most samples exhibit tensile stress, except for S13 (−1.22 ± 0.04 GPa) and SH (−0.89 ± 0.12 GPa). Both S13 and SH were deposited at room temperature. Generally, residual stress comes from intrinsic stress, extrinsic stress and thermal stress. Thermal stress is caused by the thermal expansion coefficient mismatch between the film and substrate, and it can be estimated as follows:

(11) $$\sigma_{f}\left(T\right)=\frac{E}{1-\nu_{f}}\left(\alpha_{f}-\alpha_{s}\right)\cdot \left(T_{d}-T\right)$$

where α is the coefficient of thermal expansion (CTE), E is the elastic modulus and $\upsilon_{f}$ is the Poisson’s ratio. $\left(T_{d}-T\right)$ stands for the temperature difference between the deposition temperature and room temperature. The subscript f and s represents film and substrate, respectively. The $\upsilon_{f}$ of CoCrFeNi films is estimated to be 0.275 [38]. The CTE of Si substrate is $4.0\times 10^{-6}{\;K}^{-1}$, and that of CoCrFeNi film is estimated to be 17 × 10^−6^ K^−1^ [39,40]. According to Equation (11), the values of thermal stress at 150 °C, 250 °C and 350 °C are calculated to be 0.56 GPa, 0.86 GPa and 1.23 GPa, respectively. Thermal stress developed in films at elevated temperatures upon cooling down to room temperature. The residual stress should be more tensile with increasing substrate temperature because the CTE of CoCrFeNi films is higher than that of the Si substrate, which is in accordance with laser curvature measurement results in Table 2 and Figure 7.

### 3.2. Optimization of Film Properties

#### 3.2.1. Resistivity

**Figure 8:**
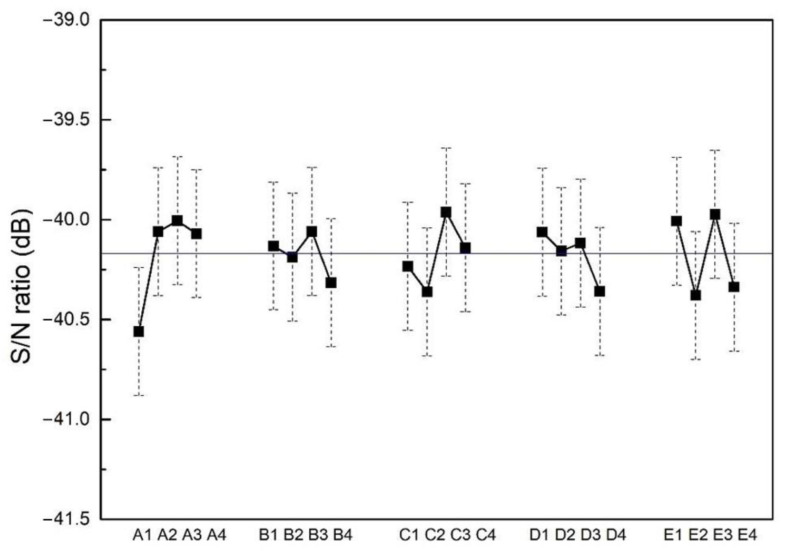
Taguchi analysis plot for film resistivity, where the experimental factors (A, B, C, D and E) and levels (1, 2, 3 and 4) can be found in Table 1. The horizontal line across the figure represents the mean S/N ratio of the 16 trials.

The resistivity of each sample measured by a four-point probe is listed in Table 2. Overall, the average resistivity is measured to be 102.1 ± 1.3 μΩ∙cm. The electrical resistivity of the 16 trials is quite similar; the largest resistivity is 112.4 ± 2.7 μΩ∙cm, and the smallest one is 97.3 ± 4.1 μΩ∙cm. The average S/N ratios of each trial are calculated by a smaller-the-better model. Figure 8 displays the average S/N ratios of each parameter level. The solid square represents the average S/N ratio at each parameter level, while the vertical dash line represents the 95% confidence interval of S/N ratios at each parameter level. The horizontal line across the figure represents the mean S/N ratio of the 16 trials. The results in Figure 8 present that substrate bias is the most sensitive parameter for electrical resistivity. To further analyze how different factors affect the electrical resistivity of films, an ANOVA analysis is performed. According to the results in Table 4, the substrate bias (38.07%) and pulsed frequency (25.18%) have significant roles in tailoring the value of resistivity. Based on a Taguchi analysis, the optimized parameters for low resistivity of CoCrFeNi films are substrate bias = −100 V, substrate temperature = 250 °C, working pressure = 1.1 mTorr, rotation = 11 rpm and pulsed frequency =120 kHz (A3, B3, C3, D1 and E3). The confirmed trial (SE) shows that the predicted S/N ratio at an optimized parameter was calculated to be 5.4 ± 4.8 dB, and the corresponding roughness is 0.5 ± 0.6 nm. The electrical resistivity of SE is 98.2 ± 0.7 μΩ∙cm, which is within the expected range. The resistivity (98.2 ± 0.7 μΩ∙cm) in this study is lower than those reported in the literature, such as nanotwinned 330 stainless steel sputtered films (110 μΩ∙cm) [41] and nanotwinned CoCrFeNi sputtered films (113.7–135.1 μΩ∙cm) [42]. Thus, the Taguchi analysis is effective for optimizing the electrical resistivity of thin films.

#### 3.2.2. Surface Roughness

**Figure 9:**
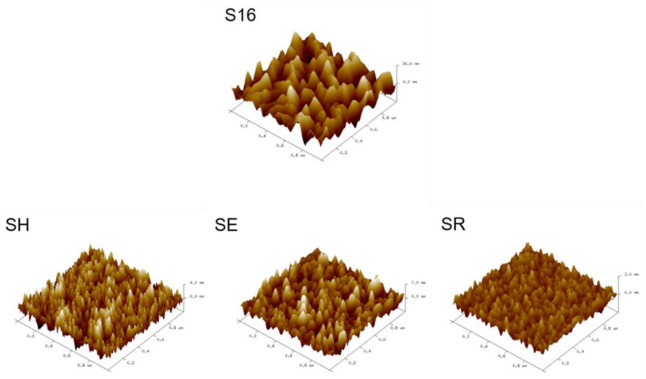
SPM surface topographies of some specimens (S16, SH, SE and SR).

**Figure 10:**
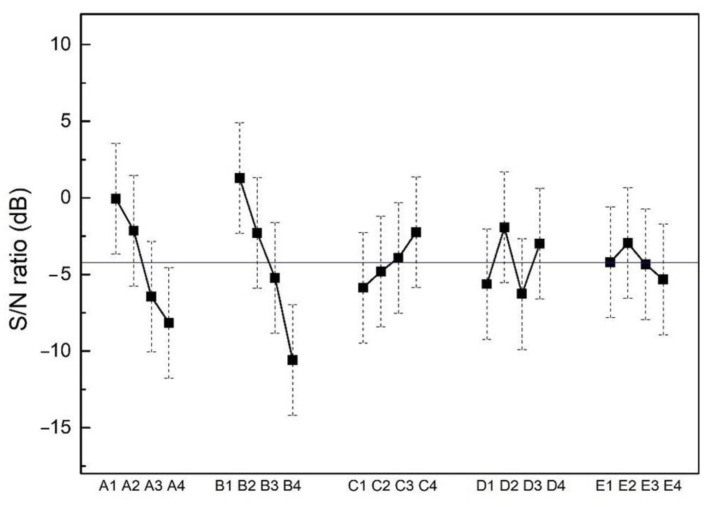
Taguchi analysis plot for film roughness, where the experimental factors (A, B, C, D and E) and levels (1, 2, 3 and 4) can be found in Table 1. The horizontal line across the figure represents the mean S/N ratio of the 16 trials.

The surface roughness of each film measured by SPM is listed in Table 2, and the 3D topography images of some samples are displayed in Figure 9. The average film roughness of the 16 samples is 2.1 ± 0.3 nm. Figure 10 demonstrates the S/N ratios of each parameter level calculated by a smaller-the-better model. It indicates that the surface roughness increases significantly with both bias and substrate temperature and increases slightly with working pressure. The abovementioned observation can be further quantified by ANOVA analysis. Based on the ANOVA results shown in Table 5, substrate temperature (53.74%) has a significant impact on roughness, followed by substrate bias (29.96%). A higher temperature may facilitate the growth of grain size and probably further enhance the surface roughness due to thermal grooving. However, a single-variable experiment should be conducted to further understand the underlying mechanism. On the other hand, rotation speed and pulsed frequency have a slight influence on roughness. The optimized parameters for a low surface roughness of CoCrFeNi film can be predicted as substrate bias = no bias, substrate temperature = room temperature, working pressure = 0.9 mTorr, rotation = 24 rpm and pulsed frequency = 60 kHz (A1, B1, C4, D2 and E2). The predicted S/N ratio at the optimized parameter was calculated to be 5.4 ± 4.8 dB, and the corresponding roughness is 0.5 ± 0.6 nm. In order to verify the Taguchi analysis, the confirmed trial (SR) was deposited under such conditions, and the confirmed test (SR) shows that surface roughness is 0.5 ± 0.0 nm. The surface roughness (0.5 ± 0.3 nm) in this study is notably lower than those reported in the literature, such as sputtered AlCoCrCu_0.5_FeNi films (2.64–25.2 nm) [24] and CoCrFeNi sputtered films (1.91–3.88 nm) [42].

#### 3.2.3. Mechanical Strength

**Figure 11:**
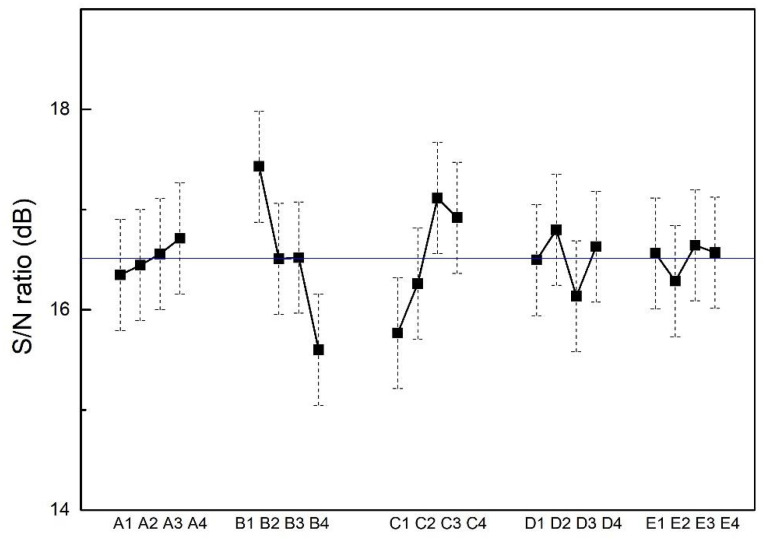
Taguchi analysis plot for film hardness, where the experimental factors (A, B, C, D and E) and levels (1, 2, 3 and 4) can be found in Table 1. The horizontal line across the figure represents the mean S/N ratio of the 16 trials.

Table 2 shows the hardness measured by nanoindentation. Overall, the average hardness of the 16 trials is 7.0 ± 0.1 GPa, and Young’s modulus is 207.8 ± 4.4 GPa. The S/N ratios were calculated by a larger-the-better model to find the optimal parameters for superior hardness. According to the S/N ratio results shown in Figure 11, the film hardness decreases with substrate temperature substantially, which may be attributed to the greater grain size enhanced by elevated temperature. In addition to substrate temperature, working pressure also affects film hardness. When working pressure is low, the deposited atom with a higher energy tended to strike the substrate strongly, resulting in a denser film with compressive stress [24].

The result based on ANOVA analysis is shown in Table 6. The substrate temperature has a great impact (52.15%) on film hardness, followed by working pressure (35.78%), whereas bias, rotation speed and frequency are less influential on film hardness. From the S/N ratio analysis, the optimized parameters for the high hardness of CoCrFeNi films can be predicted as substrate bias = −150 V, substrate temperature = room temperature, working pressure = 1.1 mTorr, substrate rotation speed = 24 rpm and pulsed frequency = 120 kHz (A4, B1, C3, D2 and E3). The predicted S/N ratio of films deposited at optimized conditions was calculated to be 18.0 ± 1.4 dB, and the corresponding value is 8.0 ± 1.2 GPa. The confirmation experiment (SH) shows that the film SH has the highest hardness of 9.3 ± 0.2 GPa, being close to the prediction value.

**Figure 12:**
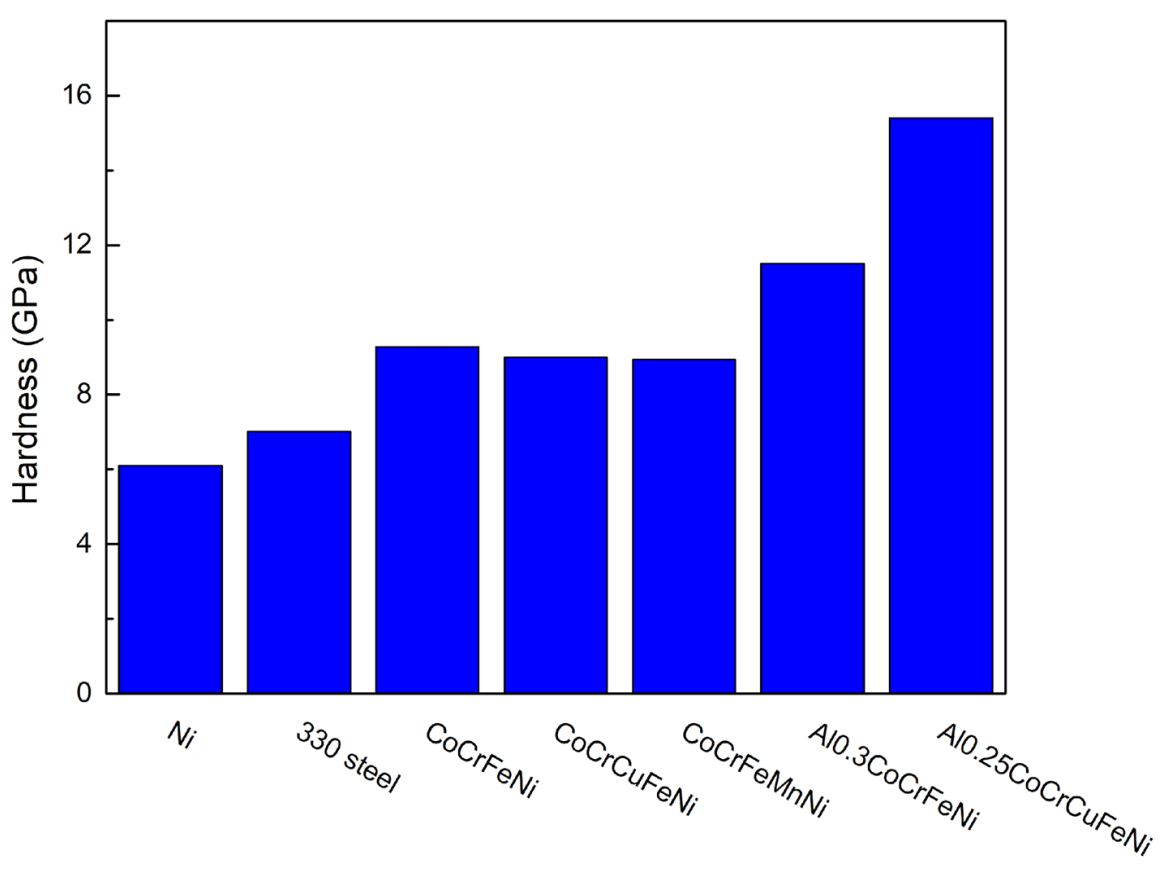
Hardness of Ni [28], 330 stainless steel [26], CoCrFeNi [this work], CoCrCuFeNi [29], CoCrFeMnNi [11], Al_0.3_CoCrFeNi [30] and Al_0.25_CoCrCuFeNi thin films [31].

Figure 12 shows the hardness of Ni [43], 330 stainless steel [41], CoCrFeNi (this work), CoCrCuFeNi [44], CoCrFeMnNi [25], Al_0.3_CoCrFeNi [45] and Al_0.25_CoCrCuFeNi thin films [46]. It is observed that the hardness of CoCrFeNi is considerably higher than that of Ni film and 330 stainless steel film and is comparable with other CoCrFeNi-based high entropy alloys. The hardness (9.3 ± 0.2 GPa) in this study is similar to other CoCrFeNi sputtered films [23,42].

## 4. Conclusions

A Taguchi L_16_ (4^5^) orthogonal array was chosen to investigate the influence of the experimental parameters on the electrical resistivity, surface roughness and film hardness. Five deposition parameters, including substrate bias, substrate temperature, working pressure, rotation speed of substrate and pulsed frequency, were set to four levels.

1. Among these five factors, substrate bias was the most sensitive factor to electrical resistivity, whereas substrate temperature was dominant in terms of influencing the surface roughness and hardness, which may be attributed to the greater grain size enhanced by elevated temperature.
2. The optimized parameters for low electrical resistivity (98.2 ± 0.7 μΩ∙cm), low surface roughness (0.5 ± 0.0 nm) and high hardness (9.3 ± 0.2 GPa) were achieved.
3. Dual FCC and HCP phases were found locally in CoCrFeNi thin films, because the energy provided from the sputtering system and additional applied substrate bias may facilitate the atoms to overcome the barrier of FCC-HCP phase transformation.

## Figures and Tables

**Table 1:** Experimental factors and levels.

Levels	Experimental Factors				
	A	B	C	D	E
	Bias (V)	Substrate Temperature (°C)	Working Pressure (mTorr)	Rotation Speed of Substrate (rpm)	Pulsed Frequency (kHz)
1	0	RT	1.6	11	20
2	−50	150	1.4	24	60
3	−100	250	1.1	34	120
4	−150	350	0.9	43	250

**Table 2:** Experimental orthogonal arrays and summary results of each thin film.

No.	A	B	C	D	E	Thickness (nm)	Deposition Rate (nm/min)	Average Grain Size (nm)	FWHM of Each Phase		Lattice Parameter (Å)	Resistivity (μΩ·cm)	Roughness (nm)	Residual Stress (GPa)	Hardness (GPa)
									(111)	(200)					
S1	1	1	1	1	1	1051.9 ± 10.1	13.1 ± 0.1	8.3	0.37	1.44	3.57	103.5 ± 2.9	0.8 ± 0.1	0.32 ± 0.03	6.9 ± 0.1
S2	1	2	2	2	2	1122.0 ± 8.7	14.0 ± 0.1	6.7	0.67	1.47	3.58	111.6 ± 2.4	0.6 ± 0.1	0.35 ± 0.03	6.6 ± 0.1
S3	1	3	3	3	3	1082.9 ± 13.8	13.5 ± 0.2	9.1	0.76	2.65	3.57	99.7 ± 1.9	1.4 ± 0.1	0.51 ± 0.02	7.1 ± 0.1
S4	1	4	4	4	4	1174.4 ± 13.0	14.7 ± 0.2	11.5	0.70	1.00	3.58	112.4 ± 2.7	1.6 ± 0.2	0.43 ± 0.01	6.5 ± 0.1
S5	2	1	2	3	4	1170.9 ± 12.1	14.6 ± 0.2	9.0	0.35	-	3.58	103.6 ± 2.7	1.0 ± 0.2	0.35 ± 0.03	7.2 ± 0.2
S6	2	2	1	4	3	1005.5 ± 11.3	12.6 ± 0.1	9.0	0.44	1.47	3.57	101.4 ± 3.9	1.1 ± 0.2	0.42 ± 0.01	6.5 ± 0.2
S7	2	3	4	1	2	1064.7 ± 12.0	13.3 ± 0.2	9.9	0.64	2.49	3.58	100.1 ± 1.6	1.1 ± 0.2	0.43 ± 0.02	7.0 ± 0.1
S8	2	4	3	2	1	1074.2 ± 16.7	13.4 ± 0.2	8.6	0.61	1.24	3.57	97.8 ± 2.7	2.0 ± 0.2	0.40 ± 0.04	6.9 ± 0.1
S9	3	1	3	4	2	1036.4 ± 6.1	13.0 ± 0.1	8.8	0.33	-	3.57	101.6 ± 3.5	0.8 ± 0.1	0.49 ± 0.01	8.2 ± 0.2
S10	3	2	4	3	1	947.5 ± 11.0	11.8 ± 0.1	11.5	0.41	1.13	3.57	97.3 ± 4.1	1.7 ± 0.2	0.35 ± 0.02	7.1 ± 0.3
S11	3	3	1	2	4	1006.4 ± 8.3	12.6 ± 0.1	10.2	0.58	1.40	3.56	101.1 ± 4.3	2.5 ± 0.3	0.74 ± 0.05	6.7 ± 0.2
S12	3	4	2	1	3	1011.2 ± 10.9	12.6 ± 0.1	15.6	0.52	1.10	3.56	100.3 ± 2.5	5.6 ± 0.7	0.78 ± 0.02	6.2 ± 0.2
S13	4	1	4	2	3	994.6 ± 2.9	12.4 ± 0.1	8.1	0.40	-	3.58	97.5 ± 3.7	0.8 ± 0.1	−1.22 ± 0.02	8.7 ± 0.2
S14	4	2	3	1	4	913.3 ± 8.9	11.4 ± 0.1	13.1	0.33	-	3.57	99.1 ± 1.9	2.6 ± 0.3	0.14 ± 0.04	7.6 ± 0.2
S15	4	3	2	4	1	957.1 ± 10.1	12.0 ± 0.1	10.9	0.39	1.46	3.56	101.9 ± 2.7	2.6 ± 0.4	0.67 ± 0.01	7.0 ± 0.1
S16	4	4	1	3	2	974.0 ± 8.0	12.2 ± 0.1	12.9	0.44	0.83	3.56	104.9 ± 3.3	7.1 ± 0.5	0.98 ± 0.07	5.5 ± 0.2
SH	4	1	3	2	3	945.0 ± 11.5	11.8 ± 0.1	6.7	0.36	1.46	3.57	100.1 ± 2.3	1.0 ± 0.1	−0.89 ± 0.05	9.3 ± 0.2
SE	3	3	3	1	3	959.2 ± 4.4	12.0 ± 0.1	7.0	0.48	1.82	3.58	98.2 ± 2.1	1.6 ± 0.1	0.49 ± 0.04	6.8 ± 0.2
SR	1	1	4	2	2	1036.0 ± 24.5	13.0 ± 0.3	8.5	0.36	1.66	3.58	105.1 ± 1.9	0.5 ± 0.1	0.12 ± 0.03	8.1 ± 0.1

**Table 3:** Chemical composition (at.%) of each film measured by SEM-EDS.

Specimen Number	Co (at.%)	Cr (at.%)	Fe (at.%)	Ni (at.%)	O (at.%)	Sum (at.%)
S1	26.5	25.2	23.1	22.9	2.3	100
S2	26.5	26.3	23.1	22.3	1.9	100
S3	26.7	25.1	22.7	23.1	2.4	100
S4	25.9	25.8	23.1	22.7	2.5	100
S5	26.1	25.8	23.5	22.6	2.1	100
S6	26.0	25.6	23.3	22.4	2.7	100
S7	26.7	25.3	22.9	22.6	2.5	100
S8	26.9	25.4	22.9	22.9	2.0	100
S9	26.9	25.3	22.8	22.9	2.1	100
S10	26.4	26.0	23.0	22.4	2.3	100
S11	27.4	25.0	23.3	22.1	2.1	100
S12	27.5	25.3	22.6	22.2	2.4	100
S13	26.9	24.8	22.4	22.8	3.0	100
S14	27.6	25.7	22.8	22.3	1.7	100
S15	27.1	25.7	22.6	22.3	2.2	100
S16	28.4	26.0	22.6	20.9	2.2	100
SH	26.9	24.8	23.5	22.8	2.0	100
SE	27.3	24.8	23.2	22.9	1.8	100
SR	26.1	25.4	23.1	23.6	1.8	100
Average	26.8 ± 0.6	25.4 ± 0.4	23.0 ± 0.3	22.6 ± 0.5	2.2 ± 0.3	100

**Table 4:** ANOVA analysis results for film resistivity. () indicates non-dominant factor.

Factor	SS	DOF	Contribution	MS	F
Substrate Bias	0.81	3	39.55%	0.27	3.54
Substrate Temperature	0.14	(3)	(6.94%)	pooled	-
Working Pressure	0.34	(3)	(16.66%)	pooled	-
Rotation Speed	0.20	(3)	(9.96%)	pooled	-
Frequency	0.55	3	26.89%	0.18	2.40
Error	0.68	9	33.56%	0.08	-
Total	2.04	15			

**Table 5:** ANOVA analysis results for film roughness. () indicates non-dominant factor.

Factor	SS	DOF	Contribution	MS	F
Substrate Bias	168.59	3	29.96%	56.20	5.52
Substrate Temperature	302.38	3	53.74%	100.79	9.89
Working Pressure	28.24	(3)	(5.02%)	pooled	-
Rotation Speed	51.92	(3)	(9.23%)	pooled	-
Frequency	11.52	(3)	(2.05%)	pooled	-
Error	91.68	-	16.29%	10.19	-
Total	562.65	15			

**Table 6:** ANOVA analysis results for film hardness. () indicates non-dominant factor.

Factor	SS	DOF	Contribution	MS	F
Substrate Bias	0.29	(3)	(2.30%)	pooled	-
Substrate Temperature	6.67	3	52.15%	2.22	12.96
Working Pressure	4.57	3	35.78%	1.52	8.89
Rotation Speed	0.95	(3)	(7.45%)	pooled	-
Frequency	0.30	(3)	(2.32%)	pooled	-
Error	1.54	9	12.07%	0.17	-
Total	12.79	15			

## Data Availability

The data presented in this manuscript will be available from the authors on reasonable request.
